# Epidemiology of Hookworm Infection in the School-age Children: A Comparative Cross-sectional Study

**Published:** 2018

**Authors:** Berhanu ELFU FELEKE

**Affiliations:** Dept. of Epidemiology and Biostatistics, University of Bahir Dar, Bahir Dar, Ethiopia

**Keywords:** Prevalence, Hookworm, Children, Urban, Rural, Ethiopia

## Abstract

**Background:**

Globally more than 740 million peoples are infected with hookworm. In sub-Saharan Africa, approximately 200 million people have been infected with hookworm, 90 million of them were children. The objective of this study was to identify the prevalence and determinant factors of hookworm infection in urban and rural school-age children’s.

**Methods:**

This comparative cross-sectional study design was conducted in Bahir Dar and Mecha district, Bahir Dar, Ethiopia from Mar-May, 2014. Epi-info software was used to calculate the sample size. Multistage sampling technique was used to select the children’s. Blood and stool samples were collected from the children to determine the hemoglobin level and the presence of parasites. Data were entered into the computer using Epi-info software and transferred to SPSS for analysis. Descriptive statistics were used to identify the prevalence of hookworm and binary logistic regression was used to identify the determining factors for hookworm.

**Results:**

The prevalence of hookworm was 22.3% [21%–24%]. Hookworm infection was associated with gender (AOR 1.31, 95% CI [1.03–1.66]), wearing shoe (AOR 0.35, 95% CI [0.25–0.48]), hand washing practice (AOR 0.62, 95% CI [0.48–0.79]), personal hygiene (AOR 0.45, 95% CI [0.34–0.61]), age (AOR 0.44, 95% CI [0.34–0.57]) or availability of latrine (AOR 0.08, 95 % CI [0.06–0.1]). Hookworm infection significantly decreases the school performance of children.

**Conclusion:**

High prevalence of hookworm infection was observed. The ministry of health and ministry of education should include deworming activity as one strategy to increase quality of education.

## Introduction

Hookworm is a parasite group called nematode living in the small intestine of humans, dogs or cats. Hookworm has two main species called *Ancylostoma duodenale* and *Necator americanus*. The latter is the predominant etiologic agent for hookworm infection (1, 2). Hookworm is one of the neglected tropical diseases listed by WHO. Neglected tropical diseases resulted in 46–57 million disability-adjusted life years lost and they account the top 4^th^ leading cause of communicable diseases (3).

Each year 3000–6500 people die due to hookworm. Hookworm hinders the immune function of the individuals and increases the risk of acquiring diseases such as HIV/AIDS, seizures, portal hypertension and diarrhea. Hookworm infection is the leading cause of anemia in sub-Saharan Africa. *A. duodenale* ingests around 150 mL of blood per a day; *N. americanus* ingests 30 mL of blood per day. Hookworm can leave permanent sequel on cognitive performance and growth of children’s, hookworm decreases the school performance of children by 20 % (3, 4).

In sub-Saharan Africa, more than 200 million people have been infected with hookworm and 90 million of them were children. In Ethiopia, more than 11 million peoples were infected with hookworm, the third highest burden in sub-Saharan Africa (5, 6). The prevalence of hookworm in southern part of the country ranges from 14.6%–50.45%, in the middle of awash valley 53% of the school children’s were infected with hookworm. Only 6.7% of the school children in Babile were infected with hookworm (7, 8). In northwest Ethiopia, the prevalence of hookworm among school-age children’s was 9.4% (9). In Fasilides elementary school in Gondar, the prevalence of intestinal parasite in elementary school children’s was 72.9%, the prevalence of hookworm was 8.2% (10). In Zarima town northwest Ethiopia, 19% of the school children’s were infected with hookworm (11). In Adwa elementary school children, the prevalence of intestinal parasite among school children’s was 69% (12). *N. americanus* is the etiologic agent for hookworm infection in Ethiopia and in Amhara region (13).

The risk of hookworm infection differs with age, gender, availability of latrine, wearing shoes, level of education, level of mothers’ education, low household income, poor personal hygiene, hand washing practice, residence, source of water (4, 14).

Intervention against hookworm in Ethiopia neither addressed well nor used the effective intervention strategies to curb the infection in the local context. This study will help the high-level decision makers to know the burden of hookworm and to choose the effective prevention strategies for hookworm in the local context. Moreover, this study will give important information about the effect of hookworm on the school performance of the children’s.

The objective of this study was to identify the prevalence and determinants of hookworm infection in urban and rural school age children’s and this aim was achieved.

## Methods

This comparative cross-sectional study was conducted in the city of Bahir Dar and Mecha district. The city of Bahir Dar located at geographical coordinates of 11° 38’ north latitude and 37° 15’ east longitude. Bahir Dar city contains 37 elementary schools with a total of 45740 students. Mecha district is a rural area located 40 km to the north of Bahir Dar with 40 kebeles (the smallest administrative unit in Ethiopia). The district contains 101 elementary schools and 80727 children’s inside these elementary schools. The target population was all elementary school-age children’s.

The study population was selected elementary school students from Bahir Dar and Mecha district. The sample size was calculated using Epi-info software version 7 assuming 95% CI, 90% power, 50% outcome in the exposed group, odds ratio of 1.5, design effect of 1.8, a rural to urban ratio of 2 and a 15% non-response rate. The final sample size was 2509 students. A multistage sampling technique was used. For Bahir dar, first 10 elementary schools were selected from a total of 37 elementary schools using lottery method. Then simple random sampling technique was used to select the children’s from 10 schools. For Mecha district: first 9 Kebele’s were selected from a total of 40 kebeles using lottery methods, one elementary school was selected from each kebeles using simple random sampling techniques then simple random sampling techniques were used to select the required number of school children from 9 schools. The data were collected from Mar–May, 2014. The data collection process contains two parts, the first part was interviewing the parents/caretakers of the children’s and the second part was collecting the stool and blood samples from the children’s. For the interview part first, the questioner was prepared in English then translated to Amharic (local language) then back to English to keep its consistency. The interview was conducted by 25 diploma nurse professionals and supervised by 5-degree holder health professionals. The blood and stool samples were collected by 8 first degree holder laboratory technologists and supervised by 2-sec degree holder laboratory technologist. From each student, one gram stool sample was collected in 10 ml SAF (sodium acetate-acetic acid-formalin solution). A concentration technique was used. The stool sample was well mixed and filtered using a funnel with gauze then centrifuged for one minute at 2000 RPM (revolution per minute) and the supernatant was discarded. Overall, 7 mL normal saline was added, mixed with a wooden stick, 3 ML ether was added and mixed well then centrifuged for 5 min at 2000 RPM. Finally, the supernatant was discarded and the whole sediment was examined for parasite. One mL blood sample was collected from children following standard operational procedures to measure the hemoglobin level of children using Mindray hematology analyzer.

To maintain the quality of the data pretest was conducted in 50 parents, training was given for data collectors and supervisors and the whole data collection process was closely supervised by the investigator and supervisors. The collected data were checked for its completeness. The data were entered in the computer using Epi-info software by 10 IT (information technology) technicians and analyzed by using SPSS software ver. 20 (Chicago, IL, USA).

Descriptive statistics were used to identify the prevalence of hookworm, two samples t-test were used to identify the effect of hookworm on school performance of the children or hemoglobin count of the children’s. Binary logistic regression (backward) was used to discover the determinants of hookworm infection, variables with *P*-value less than 0.05 and 95% CI were used to discover the determinants of hookworm infection.

Ethical clearance was granted from Amhara National Regional State Health Bureau Ethical Committee. Permission was obtained from the school directors, written consent was obtained from parents/caretakers of the children’s. The confidentiality of the data was kept at all steps. Study participants the right to withdraw from the study at any point was respected. Students with intestinal parasites or low hemoglobin counts were referred to the nearby health center for further management.

## Results

### Hookworm in rural elementary school students

Overall, 1588 rural elementary school students were included with a response rate of 94.92%. Male students constitute 52.1%, 78.5% of the students were barefooted during the time of interview. The mean age of the students was 12.65 yr (SD 2.89 yr), the mean hemoglobin count of the students was 11.45 g/dl (SD 0.71 g/dl) and their previous semester cumulative average was 68.2 (SD 13.44).

### Hookworm in urban elementary school students

Totally, 802 urban elementary school students were included with a response rate of 95.93%. The mean age of the students was 13.01 yr (SD 3.1 yr). Half of the students were females and only 10.5% of the students were barefooted during the data collection period. The mean hemoglobin level of the students was 11.98 gm/dl (SD 0.59 gm/dl). The prevalence of hookworm was 10% [95% CI, 8%–12%].

### The prevalence and predictors of hookworm in urban and rural elementary school students

Overall, 2390 students were included with a response rate of 95.27%. The mean age of the students was 12.79 yr (SD 2.96 yr). More than half of the study participants (51.4%) were male. Half of the mothers of the students (1195) were illiterate. The prevalence of hookworm was 28.6% (95 % CI=26%–31%).

Hosmer and Lemshow goodness of fit test verified that the model was good at a *P*-value of 0.26. The overall prevalence of hookworm was 22.3% [95% CI=21%–24%]. Hookworm infection was associated with gender, wearing the shoe, hand washing practice, personal hygiene, age or availability of latrine. Whereas hookworm infection was not associated with mothers education, swimming habit, residence or income (Table 1).

**Table 1:** The distribution and determinants of hookworm in urban and rural elementary school children’s (n=2390)

***Variable***		***Hookworm***		***COR [ 95 % CI]***	***AOR [ 95 % CI]***	***P-value***
		***Yes***	***No***			
Wearing shoe	Yes	108	951	0.24 [0.19–0.31]	0.35[0.25–0.48]	<0.01
	No	426	905			
Hand washing practice	Yes	263	1319	0.4 [0.32–0.48]	0.62 [0.48–0.79]	<0.01
	No	271	1319			
Personal hygiene	Kept	103	879	0.27 [0.21–0.34]	0.45 [0.34–0.61]	<0.01
	Not	431	977			
Latrine	Yes	184	1635	0.07 [0.06–0.09]	0.08 [0.06–0.1]	<0.01
	No	350	221			
Age	>=13 yr	152	860	0.46 [ 0.37–0.57 ]	0.44 [0.34–0.57]	<0.01
	< 13 yr	382	996			
sex	Female	265	897	1.05 [0.86–1.28]	1.31 [1.03–1.66]	0.03
	Male	269	959			
Residence	Urban	80	722	0.28 [0.21–0.36 ]	1.48 [1–2.17]	0.05
	Rural	454	1134			

Wearing shoe decreased the risk of hookworm infection by 65% (AOR 0.35, 95% CI [0.25–0.48]). Regular hand washing practice decreased the risk of hookworm infection by 38% (AOR 0.62, 95% CI [0.48–0.79]). Personal hygiene decreased the risk of hookworm infection by 55% (AOR 0.45, 95% CI [0.34–0.61]). The overall coverage of latrine was 76.11%. Latrine decreased the risk of hookworm infection by 92% (AOR 0.08, 95% CI [0.06–0.1]).

Students whose age less than 13 yr was risky for hookworm infection. Lower age increases the risk of hookworm infection by 56% (AOR 0.44, 95% CI [0.34–0.57]). Females had 1.31 folds higher risk of hookworm infection (AOR 1.31 95% CI [1.03–1.66]) (Table 1).

The mean hemoglobin count of children with hookworm infections was 11.31 g/dl and the mean hemoglobin count of children with no hookworm infections was 11.72 g/dl. The statistical significance of these means was approved by independent samples *t*-test (*P*>0.01).

Hookworm infection affected the school performance of children. Children with hookworm infection scores a previous semester average of 67.26 whereas children without hookworm infection scores a previous semester average of 69.76. The statistical significance of these results was approved by two independent sample *t*-tests (*P*<0.1).

## Discussion

The prevalence of hookworm in the rural elementary students was 28.6% (95% CI=26%–31%). This prevalence is higher when compared to finding from Ghana, Malaysia, and Northwest Ethiopia, Babile, Gondar (9, 10, 15, 16). This may be due to high prevalence of barefooted students in the area. The prevalence of hookworm in urban and rural setting was 22.3% [95% CI=21%–24%]. This result was higher than studies from Ghana and the national prevalence of Ethiopia (6, 16), but lower than study from Sub-Saharan Africa, southern part of Ethiopia, (17–19), similar with finding from Northwest Ethiopia (9). This difference may be due to differences distributions of hookworm predictors in these areas.

The odds of hookworm among elementary students that wear shoe regularly were 65% lower than those students that had a habit of not wearing shoe (AOR 0.35, 95% CI [0.25–0.48]). This finding agrees with findings from Sub-Saharan Africa, Southern Ethiopia, Babile and Gondar Northwest Ethiopia (10, 15). This is due to the mechanism that wearing shoe prevents the entry of hookworm to the susceptible host at its infective stage.

The odds of hookworm infection among student that had a habit of regular hand washing practice was 38% lower (AOR 0.62, 95% CI [0.48–0.79]). This finding agrees with finding from Butajira southern part of Ethiopia and Zarima town Northwest Ethiopia (11, 20). This is due to the reason that proper hand washing practice breaks the transmission mechanisms.

Personal hygiene decreases the risk of hookworm infection by 55% (AOR 0.45, 95% CI [0.34–0.61]). This finding agrees with findings from Gondar (10). This is due to the fact that proper personal hygiene will break the chain of transmission mechanism.

The overall coverage of latrine was 76.11%. Latrine decreased the risk of hookworm infection by 92% (AOR 0.08, 95% CI [0.06–0.1]). This finding agrees with finding from sub-Saharan Africa and southern Ethiopia (19). This is due to the fact that the environmental chain of transmission will be blocked by latrine utilization.

The odds of hookworm infection among students whose age less than 13 yr was 56% higher than children greater than 13 yr of age (AOR 0.44, 95% CI [0.34–0.57]). This finding agrees with finding from Malaysia, Ghana, Gondar and Zarima town Northwest Ethiopia (9, 16). The frequency of contact with the soil decreases as their age increases.

Females had 1.31 folds higher risk of hookworm infection than male (AOR 1.31 95% CI [1.03–1.66]). This finding agrees with finding from Ghana and Umolante district southern Ethiopia (16, 21) but disagree with finding from Pampanga Philippines and northwest Ethiopia (10, 14). This result may be because of variation in the distribution of the determinants of hookworm in the study areas across males and females.

The mean hemoglobin count of children with hookworm infections was 11.31 g/dl and the mean hemoglobin count of children with no hookworm infections was 11.72 g/dl. The statistical significance of these means was approved by independent samples t-test. This finding agrees with results from different parts of the world (22, 23). This is because of hookworm sucks the red blood cells of infected persons significantly

Hookworm infection affects the school performance of children. Children with hookworm infection score a previous semester average of 67.26 whereas children without hookworm infection score a previous semester average of 69.76. The statistical significance of these results was approved by two independent sample *t*-tests. This is because of the effect of hookworm on the cognitive performance and development of the brain cells.

The main limitation of this study was failure to identify acute and chronic hookworm infection but since the overall aim was to show the burden of hookworm both acute and chronic infection was added to find the prevalence of hookworm infection. Among the many strengths of the study, the use of standardized laboratory procedures decreased the subjectivity of the measurements. Further longitudinal study should be conducted to know more about the rest epidemiologic predictors of hookworm infection.

## Conclusion

High level of hookworm infection was observed. Hookworm infection was determined by wearing shoe, hand washing practice, personal hygiene, latrine utilization, sex or age. The distribution of hookworm in rural and urban elementary school students were the same. The ministry of education and ministry of health should include the deworming activity as one of the strategies to enhance the quality of education. Intervention to improve the behavior of school-age children to wear shoe should be given higher priority, especially in rural areas.
